# The influence of plant extracts on viability of ST3 and ST7 subtypes of *Blastocystis* sp.

**DOI:** 10.1186/s13099-024-00613-z

**Published:** 2024-04-03

**Authors:** Karolina Kot, Adam Michaliszyn, Elżbieta Kalisińska, Małgorzata Lepczyńska

**Affiliations:** 1 Department of Biology and Medical Parasitology, Pomeranian Medical University in Szczecin, Powstańców Wielkopolskich 72, 70-111 Szczecin, Poland; 2 Department of Medical Biology, School of Public Health, University of Warmia and Mazury, Żołnierska 14C, 10-561 Olsztyn, Poland

**Keywords:** *Blastocystis* spp., ST3, ST7, Garlic, Ginger, Horseradish, Turmeric

## Abstract

*Blastocystis* sp. is one of the most frequently detected protozoa during stool specimen examination. In the last decade, the studies about the pathogenic potential of *Blastocystis* sp. have intensified. Additionally, treatment approaches against this parasite are still disputable. The study aimed to investigate the in vitro activity of the substances of natural origin against two subtypes (ST) of *Blastocystis* sp.—ST3 and ST7. Garlic and turmeric extracts exhibited the highest inhibitory effect in relation to the ST3 viability. While horseradish and turmeric were found to be the most effective extracts to the ST7 viability. The study showed that ginger, garlic, horseradish, and turmeric extracts have potent antimicrobial activity against *Blastocystis* ST3 and ST7, with the half-maximal inhibitory concentration (IC_50_) ranging from 3.8 to 4.8 µg/ml and from 3.3 to 72.0 µg/ml, respectively, and thus may be useful in the prevention and control of *Blastocystis* infections. Additionally, this research confirmed that *Blastocystis* ST7 is more resistant to the selected plant extracts treatment than *Blastocystis* ST3 which in consequence may bring some difficulties in its eradication.

## Introduction

*Blastocystis* sp. is an obligate anaerobic protozoan transmitted via the fecal–oral route, which can colonize the human large intestine. It is estimated that over 1 billion people around the world are infected with this parasite [1, 2]. In Poland, the prevalence of this parasite is in the range of 1–23.6% [3]. The pathogenic potential of *Blastocystis* sp. is the subject of many studies around the world. Some researchers suggest that this protozoan belongs to the human gut microbiome, however, under certain conditions, its presence may cause intestinal disorders. Other researchers, on the other hand, clearly point to the parasitic nature of this protozoan [4]. For this reason, the algorithm of pharmacological treatment of blastocystosis depends on the occurrence of clinical symptoms. Patients infected with *Blastocystis* sp. who experience gastrointestinal symptoms are most often treated with antibiotics such as metronidazole, while infected patients without gastrointestinal disorders are not treated [5]. However, recent studies on animal models indicate that even asymptomatic *Blastocystis* sp. infection can cause histopathological changes in the intestines of hosts [6]. In experimental studies, the presence of vacuolar forms of *Blastocystis* sp. in the gastrointestinal tissues of infected animals caused intense inflammatory infiltrates with a predominance of single-cell leukocytes and eosinophilia [6, 7]. Moreover, some strains of *Blastocystis* sp. have been found to exert pathogenic effects by disrupting the normal intestinal microbiota of the host, which is an important counterpoint to reports suggesting the commensal nature of this protozoan [8].

For many years, the classification of *Blastocystis* sp. was based on the host from which a given strain was isolated [9]. Currently, *Blastocystis* sp. has been divided into genotypic groups (subtypes) based on the sequence of the gene encoding the small subunit of rRNA (SSU rRNA) because studies using molecular techniques have revealed significant genetic variability of *Blastocystis* sp. [10]. There are 44 recognized subtypes of *Blastocystis* sp. [11]. The division into subtypes with pathogenic potential is not possible because reports indicate that the same subtype of *Blastocystis* sp. occurs both in patients with and without intestinal symptoms [12–14]. Thirteen subtypes have been found in humans: ST1-ST10, ST12, ST14, and ST16 [15]. In Poland, only ST1-ST4, ST6, ST7, and ST9 were isolated from patients, with a clear dominance of ST3 [3].

Diet has a significant impact on the development and composition of the human large intestine microbiome. Some substances of natural origin may inhibit the development of certain organisms that may have pathogenic abilities [16, 17]. There are studies indicating that some medicinal plants contain organic compounds that help effectively eradicate parasitic infections in humans [18, 19]. This group of plants includes, among others: garlic, horseradish, and ginger. Garlic (*Allium sativum* L.) possesses many beneficial and pharmacological properties in traditional and modern medicine, such as anticancer, antioxidant, antidiabetic, immunomodulatory, antithrombotic, and antimicrobial characteristics [20, 21]. It has also anti-parasitic activity against *Blastocystis* spp., *Cryptosporidium parvum*, *Giardia lamblia*, *Entamoeba histolytica*, *Leishmania tropica*, *L. major*, *Plasmodium* spp., *Trypanosoma* spp., and *Toxoplasma gondii* [22–28]. The therapeutic properties of garlic are owed to the presence of the most bioactive and potent components, allicin and thiosulfate (sulfur-containing compounds) [14]. The exact antimicrobial mechanisms of these bioactive compounds are not yet discovered, some studies suggested that organosulfur compounds act by reacting with proteins of microbes, disrupting DNA, RNA, and protein synthesis, and also damaging the cell wall and membrane [29]. Ginger (*Zingiber officinale* Rosc.) is an important ingredient of herbal medicines used in the constipation, indigestion, vomiting, and infectious diseases treatment [30]. Studies have shown that ginger extracts have significant anti-helminthic, anti-protozoal, and anti-leech activity, as well as molluscicidal and insecticidal properties [31–33]. Ginger is composed of many bioactive compounds that contribute to its recognized biological activities. Of the 400 types of compounds found in ginger, four phenolic compounds are mainly responsible for the pharmacological effects of ginger: gingerols, shogaols, paradols, and zingerone [34]. Horseradish (*Armoracia rusticana Gaertn*.) is commonly grown and used as a spice due to the properties of its roots. It contains different isothiocyanates in its cells [35]. Horseradish root has bactericidal and antiviral properties [36]. Additionally, the natural extracts from horseradish have also antifungal and insecticidal activity [37, 38]. Turmeric (*Curcuma longa L*.) comes from the equatorial region, and in many countries, it is used to color dishes to yellow-orange. It contains curcumin, which has antibacterial and antifungal effects [39]. Curcumin has also antiparasitic properties against *Giardia lamblia*, *Leishmania* spp., *Plasmodium falciparum*, *Toxoplasma gondii*, *Schistosoma mansoni*, and *Fasciola gigantica* [40–45].

Results of some research suggest that not only antibiotics can be used to treat blastocystosis, but also the inclusion of some medicinal plants in the diet can effectively lead to the eradication of the parasite [46, 47]. Therefore, the aim of the study was to analyze the effect of extracts from four selected plants (garlic, ginger, horseradish, and turmeric) at different concentrations on the viability of *Blastocystis* sp. cells. In the study, two subtypes of *Blastocystis* sp.—ST3 and ST7 were analyzed to determine the differences in their response, if any, to the substances of natural origin. We decided to analyze ST3 because it is the most common isolated subtype in patients from Poland [3]. While, ST7 has been associated with colorectal cancer [12].

## Material and methods

### *Blastocystis* subtypes

*Blastocystis* sp. was isolated from stool samples of two symptomatic individuals. The stool samples were submitted to the parasitology laboratory for medical tests. Volunteers gave oral and written consent to the use of strains in scientific research. Under Polish Law, consent from the Bioethics Committee is not required for such research.

Genetic analysis identified these two strains as *Blastocystis* subtype 3 (ST3) and *Blastocystis* subtype 7 (ST7). ST3 was isolated from a 26-year-old female with chronic, intensive gastrointestinal symptoms and Intestinal Methanogen Overgrowth (IMO) diagnosed previously. ST7 was isolated from a symptomatic 74-year-old female who had been diagnosed with rectal cancer.

### Microscopic examination, molecular detection and subtyping

The samples were provided in a fresh state. Directly upon arrival, a small part of each specimen was cultured in previously prepared Jones’ medium supplemented with 10% horse serum (Sigma-Aldrich, Poznań, Poland) and incubated at 37 °C in anaerobic condition (pH 7.1) in tightly closed polypropylene 12-ml Falcon tubes. The xenic culture, containing gut bacteria, was subcultured every 2–3 days and screened using standard microscopy. Repeated subculture in a new medium was maintained for one month to cleanse the culture medium from fecal debris [48]. Additionally, a small part of each specimen was placed in 70% ethanol for further analysis.

Prior to DNA extraction, the samples were washed three times in phosphate-buffered saline (PBS). Genomic DNA was isolated from both stool samples using the Sherlock AX kit (A&A Biotechnology, Poland) and it was stored at − 20 °C until molecular analyses were performed. Molecular detection of *Blastocystis* sp. was achieved by a PCR method to specifically amplify a 1.8 kbp fragment of the *SSU* rRNA gene of the protozoan according to Scicluna et al. [49]. Amplification reactions (25 μl) contained PCR Master Mix Plus (A&A Biotechnology, Poland), primers BhRDr/RD5 as well as SB227 (primer for *Blastocystis* ST3) and SB155 (primer for *Blastocystis* ST7) ([50], Table 1), nuclease-free water, and template DNA. Cycling parameters were 94 °C for 3 min, and 30 cycles of denaturation, annealing, and extension at 94 °C, 59 °C and 72 °C respectively, with a final extension step of 2 min at 72 °C. PCR products (5 μl) were analyzed on 1.35% agarose gel using electrophoresis and visualized with UV light. Positive PCR products were purified using Clean-Up (A&A Biotechnology, Poland) according to the manufacturer’s recommendations. The entire procedure of DNA extraction from feces, the PCR method was described previously by Kosik-Bogacka et al. [2].

**Table 1:** Primers used in PCR analyses

Gene	Forward	Reverse
BhRDr/RD5*	ATCTGGTTGATCCTGCCAGT	GAGCTTTTTAACTGCAACAACG
SB227	TAGGATTTGGTGTTTGGAGA	TTAGAAGTGAAGGAGATGGAAG
SB155	ATCAGCCTACAATCTCCTC	ATCGCCACTTCTCCAAT

*RD5—forward, BhRDr—reverse

Sequencing on the samples subject to PCR was done once using Macrogen Humanizing Genomics Europe (Amsterdam, The Netherlands). The primers used for sequencing were RD5 (5′-ATCTGGTTGATCCTGCCAGT-3′) and BhRDr (3′-GAGCTTTTTAACTGCAACAACG-5′). The resulting chromatograms were edited and assembled using Finch TV v 1.4 (Geospiza Inc., Seattle, WA, USA). The obtained sequences were then compared to the sequences of *Blastocystis* STs, previously deposited in GenBank™ (https://www.ncbi.nlm.nih.gov/genbank/) and PUBMLST databases (http://pubmlst.org/Blastocystis/). Subtypes were identified by determining the exact match (100%) or closest similarity (99%) to all known *Blastocystis* subtypes according to the classification by Stensvold et al. [51]. The sequences were submitted to GenBank™, reported with their accession numbers PP462157 and PP462158.

### Preparation of plants extracts

Plant extracts were prepared according to publications written by Suru [52] and Abdel-Hafeez et al. [46]. Freshly peeled garlic cloves, ginger, and horseradish roots (5 g) were mixed with 100 ml of distilled water and crushed in a mortar. In the case of turmeric, 5 g powder was soaked in 100 ml of distilled water. After 48 h, the solutions were filtered through a paper filter. The solutions were diluted to obtain extracts with final concentrations: 0.01, 0.05, 0.10 and 0.50 mg/ml [47]. The aqueous extracts were stored in the dark at − 20 °C until use.

### Drug susceptibility assay

The tests were performed in vitro on a 96-well plate. Each well on the plate was filled with 0.5 ml of Jones' medium containing 100,000 morphological forms of *Blastocystis* sp. The total number of parasites was previously counted in a Bürker chamber. The first column of wells was treated as a control group therefore only 0.5 ml of Jones' medium was added; 0.5 ml of garlic extract at a concentration of 0.01 mg/ml was added to the second column; 0.5 ml of garlic extract at a concentration of 0.05 mg/ml was added to the third column; 0.5 ml of garlic extract at a concentration of 0.1 mg/ml was added to the fourth column; and 0.5 ml of garlic extract at a concentration of 0.5 mg/ml was added to the fifth column. Afterward, the plate was covered with parafilm and incubated at 37 °C. The viability of *Blastocystis* sp. was examined after 24, 48, 72 and 96 h. The cultures were stained with 0.4% Trypan blue solution according to Lepczyńska and Dzika [48] and viable, unstained cells were counted in the Bürker chamber. Analogous activities were performed with the second subtype of *Blastocystis* sp. and other extracts. Each experiment was carried out in triplicates.

### Statistical analysis

The Statistica StatSoft software (version 8.0) was used in the statistical analysis. Means of triplicate data were calculated and used as the final result. The differences between groups were calculated by non-parametric tests. The graphs were presented using Microsoft Excel 2016. GraphPad 4.0 was used for the determination of the half-maximal inhibitory concentration (IC_50_). IC_50_ values were calculated using non-linear regression analysis from dose–response curves of data obtained after direct observation at 96 h and cell counts by microscopy. In this study, *p* values of ≤ 0.05 were considered significant.

## Results

The two isolates in this study were obtained from symptomatic volunteers suffering from gastrointestinal symptoms. Under light microscopy, the fecal samples were examined and were found to be infected with vacuolar forms of *Blastocystis*. Both subtypes of cells were also successfully cultured and maintained in Jones’ medium. Based on the PCR amplification and after that on sequencing, the observed genotypes were ST3 and ST7.

### ST3

Growth profile studies were carried out to evaluate the anti-protozoal property and efficacy of plant extracts against *Blastocystis* ST3. All studied aqueous extracts at all time points and at all concentrations inhibited the development of *Blastocystis* ST3, statistically significantly reducing their number of living cells compared to the control group. Moreover, it was observed that the cells treated with extracts were smaller in size than untreated ones.

**Fig. 1:**
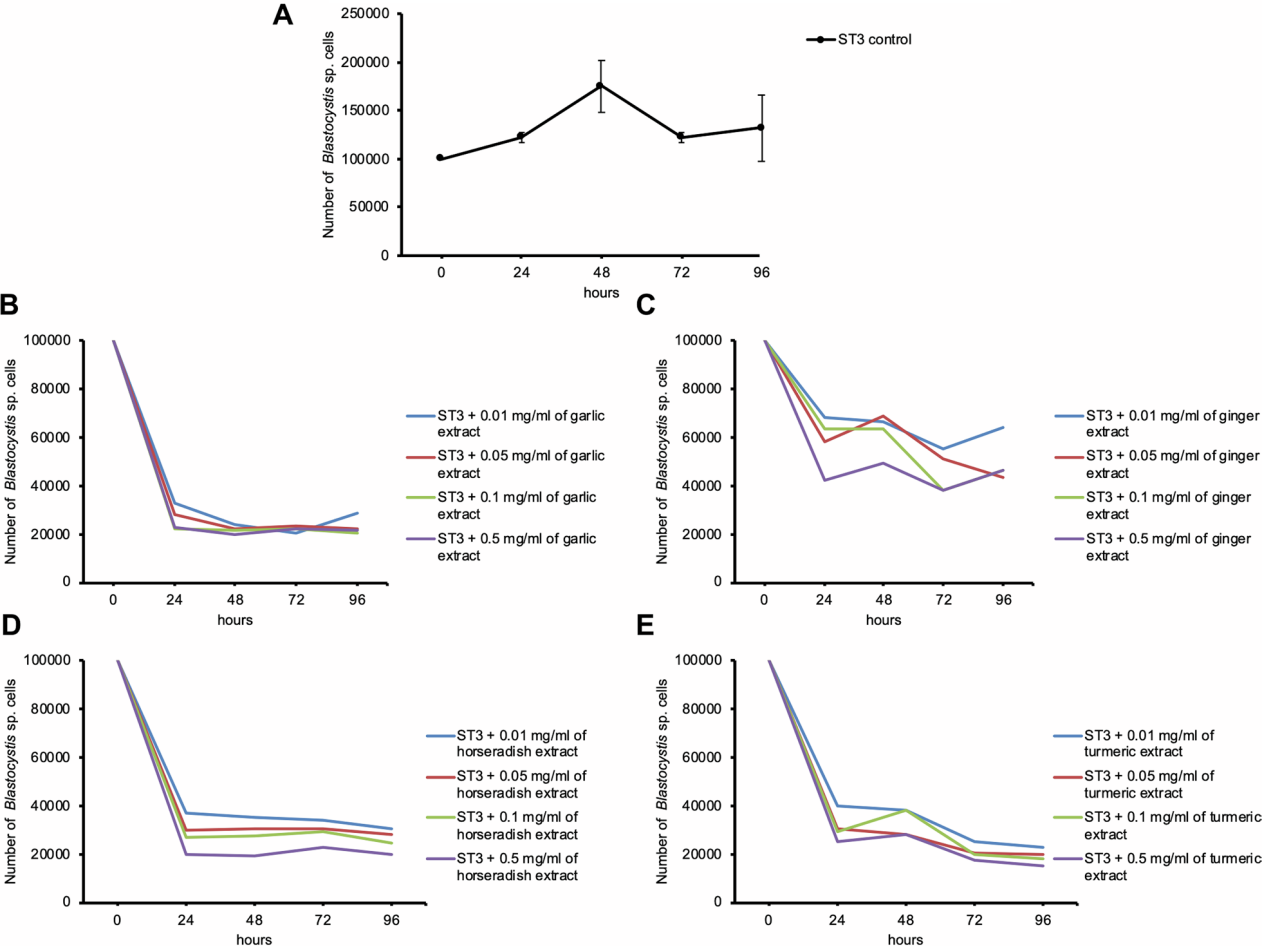
The number of *Blastocystis* subtype 3 cells in the control sample (**A**), after treatment with different concentrations of garlic (**B**), ginger (**C**), horseradish (**D**), and turmeric (**E**) extracts in various time points

The length of exposure of various aqueous plant extracts to *Blastocystis* ST3 was compared (Fig. 1). Statistically significant differences were found between 24 h vs 48 h vs 72 h vs 96 h when the microorganism was exposed to garlic extract at a concentration of 0.01 mg/ml (H = 21.17, p < 0.001) and 0.05 mg/ml (H = 15.11, p < 0.05). In the case of turmeric, significant differences were observed between time points when exposed to all turmeric concentrations: 0.01 mg/ml (H = 18.45, p < 0.001), 0.05 mg/ml (H = 12.68, p < 0.01), 0.1 mg/ ml (H = 17.88, p < 0.001) and 0.5 mg/ml (H = 15.41, p < 0.01). According to the ginger, differences were found between four-time points at average concentrations of extracts: 0.05 mg/ml (H = 14.39, p < 0.01) and 0.1 mg/ml (H = 10.45, p < 0.05). There were no statistically significant differences in horseradish concentrations between the tested time points.

Garlic extract caused significantly the highest inhibition of *Blastocystis* ST3 cell proliferation in 24 h at all tested concentrations, which was confirmed by statistical analysis. After 48 h of co-incubation, garlic extract was most effective at three concentrations: 0.01, 0.05, and 0.1 mg/ml, while horseradish extract was only at a concentration of 0.5 mg/ml. Moreover, it was found that the lowest concentration (0.01 mg/ml) of garlic extract was also most effective in the 72nd hour of co-incubation. However, at the mentioned concentrations in the 72nd hour and all studied concentrations in the 96th hour, the turmeric extract showed the greatest effectiveness.

The IC_50_ for garlic extract was 0.0039 mg/ml, for ginger extract was 0.0048 mg/ml, for horseradish extract was 0.0042 mg/ml, and for turmeric extract was 0.0038 mg/ml mg/ml.

### ST7

**Fig. 2:**
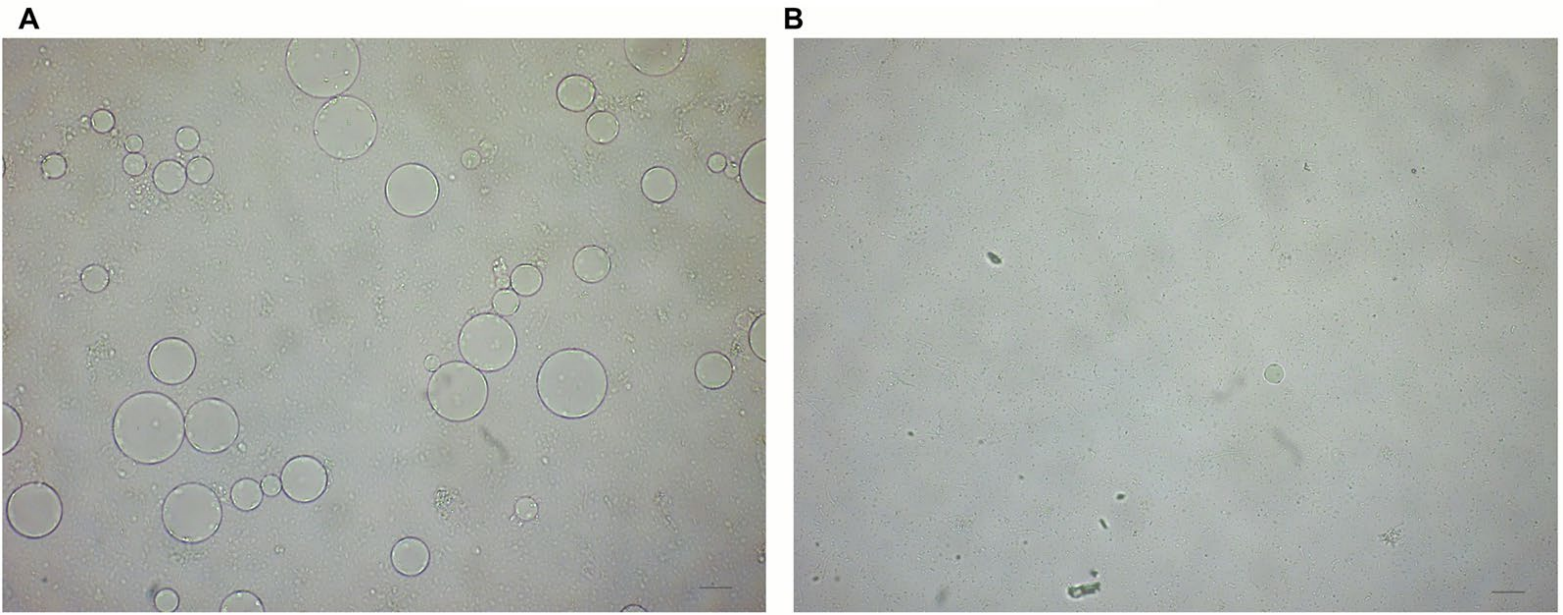
Effect of garlic extract on the growth and viability of *Blastocystis* ST7, untreated cells (**A**) and cells treated with 0.5 mg/ml after 24 h (**B**). Scale bar = 50 µm

*Blastocystis* ST7 also exhibited susceptibility to the plant extracts. At all time points and all studied extract concentrations, statistically significant differences were found between the number of *Blastocystis* ST7 cells with the added extract and the control group (without the added extract) where a lower number of *Blastocystis* ST7 cells was observed in comparison to the control group. Additionally, the cells treated with plant extracts were smaller compared to untreated ones (Fig. 2).

**Fig. 3:**
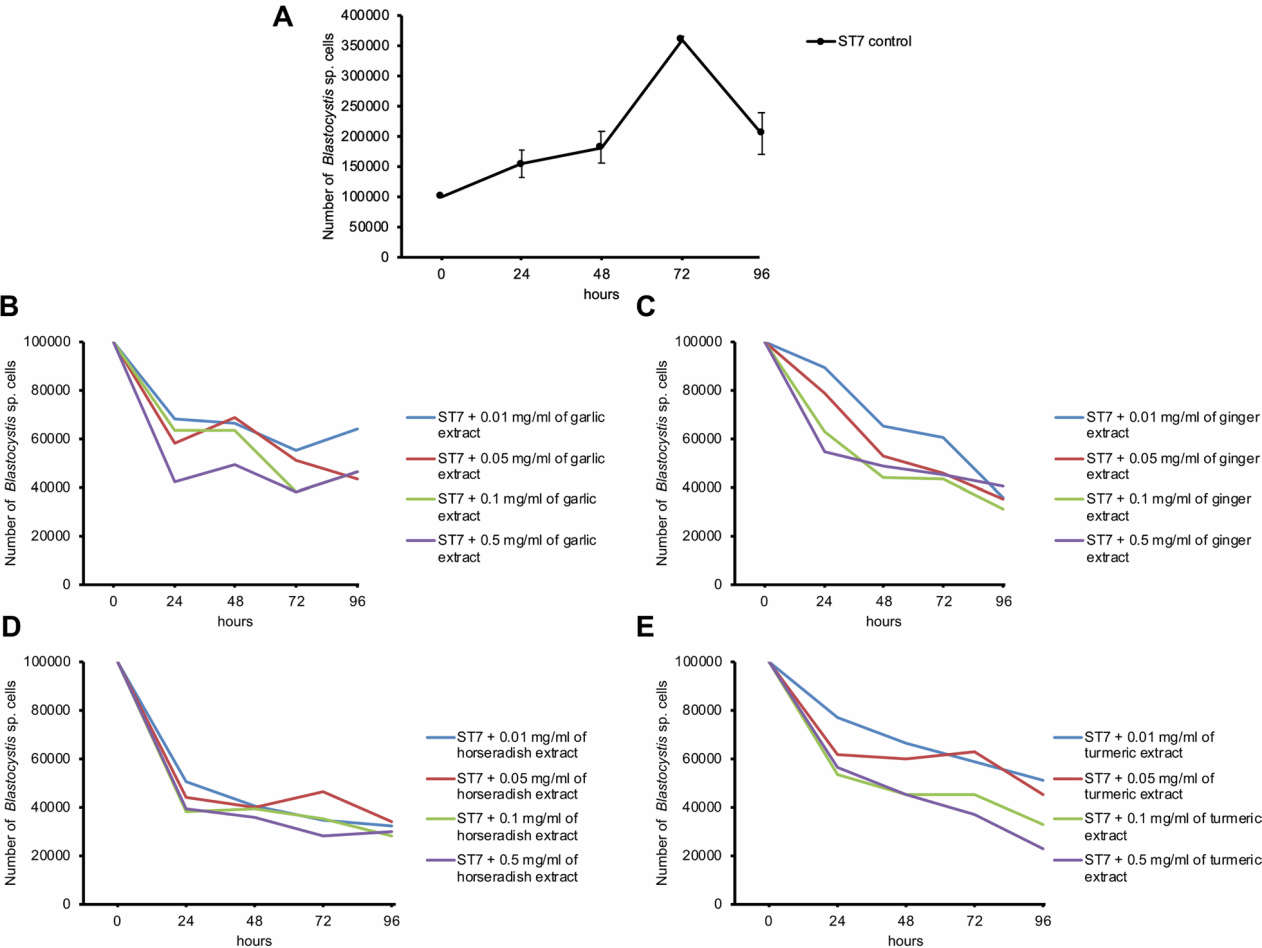
The number of *Blastocystis* subtype 7 cells in the control sample (**A**), after treatment with different concentrations of garlic (**B**), ginger (**C**), horseradish (**D**), and turmeric (**E**) extracts in various time points

The duration of exposure of *Blastocystis* ST7 to various aqueous plant extracts was compared (Fig. 3). Statistically significant differences were found between 24 h vs 48 h vs 72 h vs 96 h when protozoa were exposed to garlic extract at a concentration of 0.05 mg/ml (H = 14.39, p < 0.01) and 0.1 mg/ml (H = 10.45, p < 0.02). In the case of turmeric, significant differences were observed between time points when exposed to high concentrations of turmeric extract: 0.1 mg/ ml (H = 11.53, p < 0.01) and 0.5 mg/ml (H = 16.60, p < 0.001). When comparing horseradish and ginger, differences were found between four-time points at each tested concentration of these extracts: 0.01 mg/ml (H = 16.87, p < 0.001 and H = 19.78, p < 0.001, respectively), 0.05 mg/ml (H = 13.10, p < 0.01 and H = 21.41, p < 0.001, respectively), 0.1 mg/ml (H = 13.83, p < 0.01 and H = 19.71, p < 0.001, respectively), 0.5 mg/ml (H = 13.77, p < 0.01 and H = 9.62, p < 0.05).

Additionally, the statistical analysis was used to evaluate which of the tested extracts and at what concentration mostly inhibited cell proliferation. At 24, 48, and 72 h after treatment, the lowest number of *Blastocystis* ST7 cells was observed in the tubes with horseradish extract. At 96 h, ginger extract was most effective at concentrations of 0.01, 0.05, and 0.1 mg/ml, while turmeric extract was most effective at 0.5 mg/ml.

The IC_50_ for garlic extract was 0.072 mg/ml, for ginger extract was 0.0033 mg/ml, for horseradish extract was 0.0037 mg/ml, and for turmeric extract was 0.015 mg/ml.

## Discussion

Scientific research on *Blastocystis* sp. is increasingly focusing on a specific *Blastocystis* subtype rather than the *Blastocystis* genus because each subtype is characterized by different biochemical and pathogenic properties [53]. Some subtypes have been associated with health problems in humans as the case of ST7, while ST3 has been related as a non-pathogenic subtype [54–56]. However, it is very difficult to assign a subtype to those with pathogenic or non-pathogenic properties. It was revealed that ST7 caused disruptions in the gut epithelial barrier by influencing on tight junction proteins such as occludin and zonula occludens-1 (ZO-1), and also has greater adhesiveness than ST4 isolates to intestinal epithelial cells [57, 58]. Furthermore, ST7 was shown to have significantly greater cysteine protease activity compared to ST4 [57]. Additionally, *Blastocystis* ST7-infected patients showed a higher number of ‘harmful’ bacteria (*Proteobacteria*) in their gut. Recent data suggest that *Blastocystis* ST7 may interact with multiple members of gut microbiota, such as the *Escherichia*-*Shigella* group, to cause these negative alterations [59]. ST3, on the other hand, was associated with an eubiotic state characterized by beneficial species that are members of the phyla Firmicutes and Bacteroidetes, such as those of the genera *Ruminococcus* and *Prevotella*, respectively [60]. ST3 is mostly isolated from asymptomatic patients, however, it is also found in patients with gastrointestinal symptoms [61].

ST3 is the most dominant subtype in many countries, including highly urbanized regions [3, 62]. It is suggested that ST3 is the most human-specific subtype and is primarily transmitted among people (due to human-to-human transmission) [63]. In Poland, ST3 is the most prevalent subtype in both, males and females, in various age groups as well as in those traveling outside Europe and the patients who never left their country [2, 3]. Regarding ST7 it is considered an avian subtype because of its higher frequency in animals, including livestock, and its relative predominance in birds, both wild and farmed [64]. The highest number of ST7 human infections were reported in Nepal, Egypt, Japan, Malaysia, and Pakistan (from the highest to the lowest number of cases). In Poland, ST7 was identified much less frequently than other STs, accounting for 2.47% of cases [3]. Additionally, *Blastocystis* sp. infection has been found in patients with colorectal cancer (CRC). Sulżyc-Bielicka et al. [65] reported that CRC was associated with an increased risk of opportunistic *Blastocystis* sp. infection. The latest studies showed that subtype 7 was only isolated from CRC stool samples with significant association [12]. In this study, the volunteer with *Blastocystis* ST7 is diagnosed with rectal cancer.

The presented work assessed the effect of aqueous extract of garlic, ginger, horseradish, and turmeric on the viability of *Blastocystis* ST3 and ST7. It was found that each studied plant extract resulted in reduced ST3 viability; however, none of the studied concentrations resulted in complete eradication of the protozoan. Garlic and turmeric extracts exhibited the highest inhibitory effect in relation to the ST3 viability. The garlic activity was also observed with other protozoan parasites, such as *Giardia lamblia* [66, 67], *Cryptosporidium parvum* [68], and *Entamoeba histolytica* [23]*.* According to the literature, *Blastocystis* ST7 has been shown to be more resistant to anti-parasitic drugs [69, 70] and against the host innate immune response [71] compared to ST1 and ST4 isolates. In the present study reduced ST7 viability under the influence of each plant extract was observed, but similarly to ST3, none of the studied concentrations resulted in complete eradication of the protozoan. Horseradish and turmeric were found to be the most effective extracts in relation to the ST7 viability. Similar research was performed by Yakoob et al. [47] and Abdel-Hafeez et al. [46]. Yakoob et al. [47] found that *Blastocystis hominis* is as sensitive to metronidazole, the drug of choice for the treatment of blastocystosis, as garlic. In turn, *B. hominis* was not sensitive to ginger, black pepper, and cumin compared to garlic and metronidazole. Surprisingly, ginger appeared to be promoting the growth of *B. hominis* isolates at higher concentrations. The present study also has shown that long-term exposure to ginger extract promoted the growth of *Blastocystis* ST3, but not *Blastocystis* ST7. Based on the research of Abdel-Hafeez et al. [46] it is suggested that ginger has the greatest impact on the viability of *Blastocystis hominis*. Additionally, these authors reported that turmeric treatment insignificantly lowered the number of parasite cells. In this research, different results were observed, but it is important to note that for mentioned authors did not focus on any subtype of *Blastocystis*, only on the *Blastocystis* genus, in general.

The study showed that ginger, garlic, horseradish, and turmeric extracts have potent antimicrobial activity against *Blastocystis* ST3 and ST7, with an IC_50_ ranging from 3.8 to 4.8 µg/ml and from 3.3 to 72.0 µg/ml, respectively. The study authors found comparable IC_50_ values of anti-protozoal agents effective against *Blastocystis* ST7. Metronidazole has an IC_50_ of 31.5 μg/ml [70]. Mirza et al. [69] found that two strains within one subtype respond differently to anti-parasitic drugs. They examined nitazoxanide, furazolidone, mefloquine, quinacrine, quinine, emetine, trimethoprim sulfate-sulfamethoxazole, and iodoacetamide. The range of IC_50_ of these drugs was between 0.2 and 22.0 μg/ml [69]. This indicates that plant extracts have a potent antimicrobial effect against *Blastocystis* and are promising candidates for the alternative treatment of *Blastocystis* infections or as an additional adjunctive therapy.

The study has a limitation. We did not include a drug control group with metronidazole or nitazoxanide. The main reason for the decision was that both subtypes isolated from volunteers and currently studied were resistant to several anti-parasitic drugs, including metronidazole at the highest dose. Hence, an attempt was made to look for other alternative treatments for blastocystosis. In further studies on the effects of potential medicinal plants on *Blastocystis* sp. viability, a control trial with a drug should be added.

## Conclusions

The study confirms the necessity to analyze each of the *Blastocystis* subtypes because the differences in pathogenic and biochemical properties between them influence on the patient’s treatment results. The study showed that selected plant extracts have potent in vitro antimicrobial activity against *Blastocystis* ST3 and ST7, and thus may be useful in the prevention and control of *Blastocystis* infections. The highest inhibitory effect in relation to the *Blastocystis* ST3 viability had garlic and turmeric, while horseradish and turmeric were the most effective against the *Blastocystis* ST7. Additionally, this research confirmed that *Blastocystis* ST7 is more resistant to the selected plant extracts treatment than *Blastocystis* ST3 which in consequence may bring some difficulties in its eradication.

## Data Availability

Derived data supporting the findings of this study are available from the corresponding author (KK) on request.
